# The Central Asian Journal of Global Health to Increase Scientific Productivity

**DOI:** 10.5195/cajgh.2013.108

**Published:** 2014-01-24

**Authors:** Kyle Freese, Eugene Shubnikov, Ron LaPorte, Shalkar Adambekov, Sholpan Askarova, Zhaxybay Zhumadilov, Faina Linkov

**Affiliations:** 1 University of Pittsburgh, Pittsburgh, PA; 2 Institute of Internal Medicine, Novosibirsk, Russia; 3 Center for Life Sciences, Nazarbayev University, Astana, Kazakhstan

**Keywords:** Central Asia, scientific productivity, scientific journals

## Abstract

The WHO Collaborating Center at the University of Pittsburgh, USA partnering with Nazarbayev University, developed the Central Asian Journal of Global Health (CAJGH, cajgh.pitt.edu) in order to increase scientific productivity in Kazakhstan and Central Asia. Scientists in this region often have difficulty publishing in upper tier English language scientific journals due to language barriers, high publication fees, and a lack of access to mentoring services. CAJGH seeks to help scientists overcome these challenges by providing peer-reviewed publication free of change with English and research mentoring services available to selected authors.

CAJGH began as a way to expand the Supercourse scientific network (www.pitt.edu/~super1) in the Central Asian region in order to rapidly disseminate educational materials. The network began with approximately 60 individuals in five Central Asian countries and has grown to over 1,300 in a few short years. The CAJGH website receives nearly 900 visits per month.

The University of Pittsburgh’s “open access publishing system” was utilized to create CAJGH in 2012. There are two branches of the CAJGH editorial board: Astana (at the Center for Life Sciences, Nazarbayev University) and Pittsburgh (WHO Collaborating Center). Both are comprised of leading scientists and expert staff who work together throughout the review and publication process. Two complete issues have been published since 2012 and a third is now underway. Even though CAJGH is a new journal, the editorial board uses a rigorous review process; fewer than 50% of all submitted articles are forwarded to peer review or accepted for publication. Furthermore, in 2014, CAJGH will apply to be cross referenced in PubMed and Scopes.

CAJGH is one of the first English language journals in the Central Asian region that reaches a large number of scientists. This journal fills a unique niche that will assist scientists in Kazakhstan and Central Asia publish their research findings and share their knowledge with others around the region and the world.

